# Supplementary material for: Effect of the second chromophore energy gap on photo-induced electron injection in di-chromophoric porphyrin-sensitized solar cells
Source: R Soc Open Sci. 2018 Sep 26;5(9):181218. doi: 10.1098/rsos.181218 (PMC6170585; doi:10.1098/rsos.181218)
Supplement: Supporting Info for rsos [file rsos181218supp1.pdf]

Please do not adjust margins

Journal Name

ARTICLE

Effect of the second chromophore energy gap on photo-induced electron injection in di-chromophoric porphyrin-sensitized solar cells

Received 00th January 20xx,
Accepted 00th January 20xx

DOI: 10.1039/x0xx00000x

www.rsc.org/

Long Zhao*

This work investigates the effect of the second chromophore energy gap on charge generation in porphyrin-based di-chromophoric dye-sensitized solar cells (DSSCs). Three di-chromophoric porphyrin dyes (PorY, PorO and PorR) containing three organic chromophores with decreasing frontier orbital energy offsets, including a carbazole-triphenylamine chromophore (yellow, Y), a carbazole fused-thiophene chromophore (orange, O) or a carbazole-thiophene benzothiadiazole thiophene chromophore (red, R) were investigated using optical and electrochemical methods, steady state photoluminescence and photovoltaic device characterization. Energy transfer from the organic chromophore to the porphyrin was suggested in PorY and PorO as the main charge generation mechanism in dye-sensitized solar cells using these di-chromophoric dyes. On the other hand, electron transfer from the photo-excited porphyrin to the organic chromophore as a competing pathway leading to the loss of photocurrent is suggested for PorR-sensitized solar cells. The latter pathway leading a loss of photocurrent is due to the lower lying Lowest Unoccupied Molecular Orbital (LUMO) of the additional organic chromophore (R) and suggests the limitation of the current di-chromophoric approach to increase the overall efficiency of dye-sensitized solar cells.

Introduction

Porphyrins are one of the most remarkable sensitizers in dye-sensitized solar cells (DSSCs), achieving some of the highest power conversion efficiencies to date.[1-3] Multi-chromophoric porphyrin dyes having more than one chromophore, either increasing the extinction coefficient or extending the photon absorption wavelength range as an alternative to the co-sensitization approach, have attracted the interest of researchers to further increase the DSSC performance.[4-11] One of the interesting aspects of the multi-chromophoric design is that the electronic communication between the chromophores can be inhibited by a twisted 3D structure, weakening the dispersion forces (polarizability of the molecule) and elongating the electron lifetime.[9] Furthermore, intramolecular hole transfer between the porphyrin and the side chain chromophore can lead to faster dye regeneration rate at the TiO_2/dye/electrolyte interface without increased driving force for the electron transfer reaction.[12] However, the power conversion efficiencies (PCEs) of DSSCs using multi-chromophoric porphyrin dyes are often lower than those of the best single porphyrin or porphyrin / organic dye co-sensitized solar cell approaches. One of the best reported for example, the PCE of 6.2% was achieved by a carbazole substituted porphyrin di-chromophoric dye-sensitized solar cell,[13] which is only half the values reported using a Donor-π bridge-Acceptor (D-π-A) structured porphyrin dye (SM315)-sensitized solar cell (13%).[1]

One of the main limitations in the photovoltaic performance of reported multi-chromophoric porphyrins is that the light absorption of the second chromophore is not extended far enough beyond the porphyrin absorption. In this regard, generating suitable electronic levels in the second chromophore that lead to the extension of light absorption whilst not introducing unwanted energy / electron transfer pathways within the chromophores has been the main challenge. In principle, there are three distinct mechanisms relating to energy transfer processes. The most frequent one is Förster resonance energy transfer (FRET), which does not require strong electronic coupling between the two adjacent chromophores but is mainly restricted to singlet-singlet energy transfer.[14] A less studied but also very important energy transfer process is the Dexter mechanism, which involves electron exchange between the donor and the acceptor.[15] Though this electron transfer mechanism requires significant molecular orbital mixing of the two chromophores, it can promote both singlet-singlet and triplet to triplet excited state energy transfer.[16,17] The third possible energy transfer process is photo-induced electron transfer (PET) which causes quenching of energy by dissipating heat. A featrue of the PET quenching mechanism is the formation of exciplex $(D^{+}\bullet A^{-})^*$ after

School of Chemistry and Chemical Engineering, Jiangsu University, Zhenjiang, 212013, P. R. China; ARC Centre of Excellence for Electromaterials Science, Intelligent Polymer Research Institute, University of Wollongong, Wollongong, NSW 2522, Australia.

Electronic Supplementary Information (ESI) available: [Differential pulse voltammetry, spectro-electrochemical spectra, photoluminescence, absorbed photon-to-current conversion efficiency, photovoltaic performance, experimental section.]. See DOI: 10.1039/x0xx00000x

This journal is © The Royal Society of Chemistry 20xx

Please do not adjust margins

Please do not adjust margins

photo-excitation of the donor.[18,19]

Understanding charge generation mechanism in multi-chromophoric sensitizers is quite important in the field of multi-chromophoric dye-sensitized solar cells.[6,20] The quantum yield of these possible energy / electron transfer reactions in a di-chromophoric dye can affect the electron injection efficiency into the semiconductor. In a TiO_2-D1-D2 di-chromophoric dye sensitized film system, where D1 is proximate to the surface of TiO_2, the main route for charge generation after photo-excitation of D2 is energy transfer from D2* to D1, forming D1*, followed by electron injection from D1* to TiO_2. Similar energy transfer pathway was illustrated in artificial light-harvesting antenna macromolecules.[21] In two Re-Ru superamolecules, it is also possible for a remote electron injection from D2* to TiO_2.[22] However, one special case is that in which the Lowest Unoccupied Molecular Orbital (LUMO) of D1 lies higher than that of D2 and D1 is photo-excited. As such, the direction of energy / electron transfer is reversed under photo-excitation either due to a change in the overlap of photoluminescence and absorption spectra, or a change in the driving force for electron transfer. Energy and electron transfer from D1* to D2 may occur and decrease the electron injection yield from D1* to TiO_2 conduction band due to a kinetic competition between the two “opposite” pathways. Here in this work, steady-state photoluminescence measurements and photovoltaic devices characterization are correlated to provide information on charge transfer mechanisms in di-chromophoric dye-sensitized solar cells.

Fig.1 displays the structures of Por, PorY, PorO, PorR and the organic chromophores. The three organic chromophores employed are with systematically lower energy gaps: PorY contains a carbazole chromophore, PorO contains a carbazole-fused thiophene chromophore and PorR contains a carbazole-thiophene benzothiadiazole thiophene chromophore. The organic chromophores Y, O and R absorb photons at different wavelength range: Y for yellow, O for orange and R for red, respectively. The organic chromophore and the porphyrin were linked via a phenylethenyl unit to prevent effective electronic communication between the two chromophores by a dihedral angle.

Experimental

Differential Pulse Voltammetry (DPV) was measured with a three-electrode system using an eDAQ (BVI) instrument. The working electrode was a Pt wire with the surface area of 6.6 mm^2, the counter electrode was a slice of Pt mesh and the reference electrode was an inhouse-made Ag/AgCl wire. The Ag/AgCl potential was calibrated using 1 mM freshly prepared ferrocene (Fc, 98%, Aldrich) in the same solution.

CbTPA and PorY were measured in dichloromethane (DCM, 99.8%, Chem-supply) with concentration of 0.5 mM, while CbTh, PorO, CbBTD and PorR were measured in dimethylformamide (DMF, 99.99%, Honeywell) with concentration of 0.5 mM. Tetrabutylammonium perchlorate (TBAP, 99.0%, Fluka) was dissolved in solutions with concentration of 0.1 M as supporting

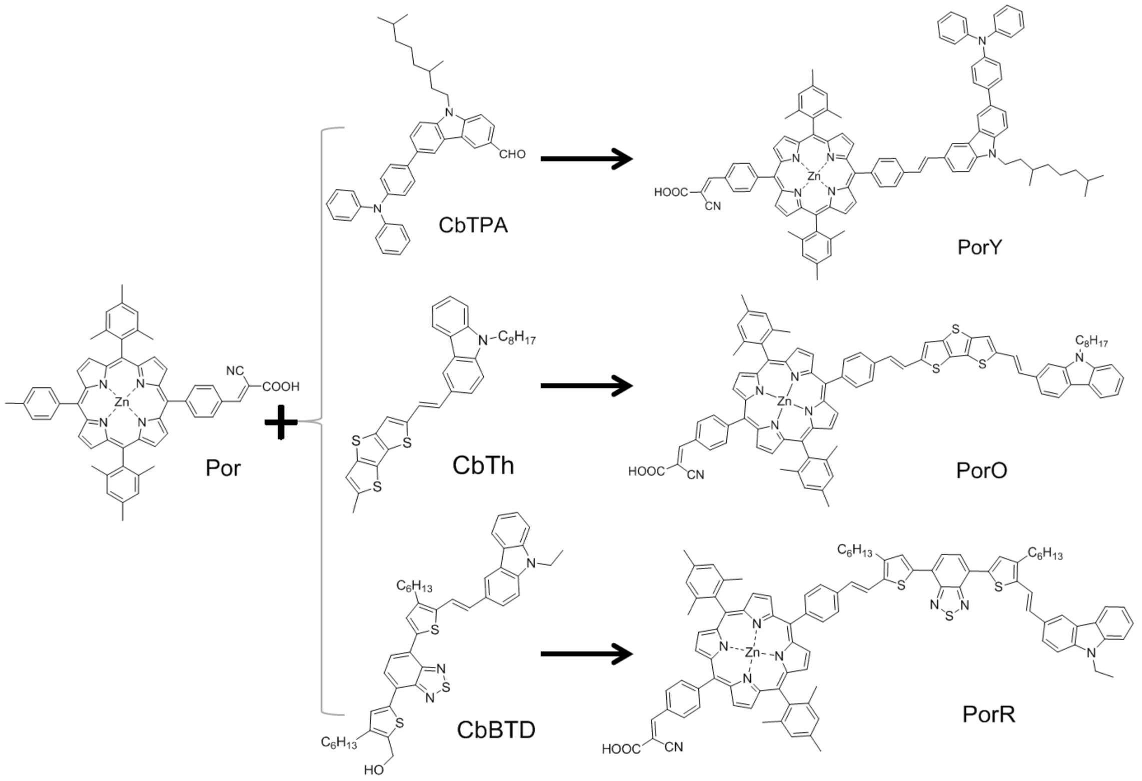

Figure 1. Molecular structures of the compounds

This journal is © The Royal Society of Chemistry 20xx

Please do not adjust margins

Please do not adjust margins

Journal Name

ARTICLE

electrolyte. Both DCM and DMF were further dried by going through a column of activated alumina and purged with argon for 30 minutes prior to use. DPV plots of the single porphyrin Por were recorded in both solutions for comparison.

UV-visible (UV-vis) absorption spectroscopy was detected using a Shimadzu UV-3600 spectrophotometer. Solutions in DMF with concentration of 1 μM were used for all compounds.

Photoluminescence spectroscopy was recorded with a fluorescence (Fluorolog® FL3-221, Horiba) spectrometer and was detected using a single photon counting interface (FluoroHub) at room temperature. Most of the compounds were performed in DMF, except for CbBTD and PorR which were measured in tetrahydrofuran (THF, 99.99%, Honeywell) with concentration of 1 μM. The slit width for emission measurement was chosen 3 nm to get modest numbers of Counts Per Second (CPS).

Dye-sensitized solar cells (DSSCs) fabrication

Thin films with mesoporous TiO_2 (18-NRT, Dyesol) thickness of 2-2.6 μm and the Pt coated counter electrodes were fabricated following the reported procedure.[13] The active surface area was 4 mm × 4 mm. The TiO_2 films were immersed into a dye solution with concentration of 0.2 mM in THF for 1.5 hours to achieve dye-sensitization. The photoanode, a Surlyn® gasket and counter electrode were sandwiched; electrolyte consists of 0.6 M 1,2-dimethyl-3-propylimidazolium iodide (DMPII, synthesized in house), 0.1 M LiI (99.9%, Aldrich), 0.05 M I_2 (99.99%, Aldrich), 0.5 M tert-butylpyridine (tBP, 96%, Aldrich) in acetonitrile (AN, 99.8%, Sigma-Aldrich): valeronitrile (VN, 99.5%, Sigma-Aldrich) with a v/v ratio of 85:15 was injected using a vacuum pump.

Current density-voltage (J-V) measurements were performed using a simulated 100 mW cm^{-2} AM 1.5G solar simulator (TriSOL, OAI) coupled to a Keithley 2400 source measure unit. A 20 minutes light soaking and three times J-V measurements were applied to the finished devices before the last recording. All the measurements used a 6 mm × 6 mm mask to set the illumination area.

Incident photon-to-current conversion efficiency (IPCE) and **Light harvesting efficiency (LHE)** were tested after the J-V measurements. IPCE measurement used a QEX10 quantum efficiency measurement system (PV measurements), which sets the beam dimensions to be smaller than the active area of the devices.[8] LHE was calculated from the UV-vis absorbance of the sensitizers on TiO_2 films with a small amount of electrolyte injected.[6,23] **Absorbed photon-to-current conversion efficiency (APCE)** was calculated according to the following equation:

$$APCE(\lambda) = \frac{IPCE(\lambda)}{LHE(\lambda)}$$

Results

Differential pulse voltammetry (DPV) of all the compounds is shown in Fig.2. Those of CbTPA and PorY were reported in Ref.[13], while those of Por, CbTh and PorO were reported in Ref.[8]. The DPV plots of CbBTD and PorR is the first time reported here.

For the organic chromophores CbTPA, CbTh and CbBTD, null to two oxidation and reduction peaks are observed. CbTPA shows two oxidation reactions while none reduction in the potential window. CbTh and CbBTD both show one broad oxidation peak at a similar potential, whereas only CbBTD displays an intense reduction peak at around -1.65 V vs Fc/Fc^+. The porphyrins, on the other hand, typically show two oxidation and two reduction peaks, except for Por and PorO measured in DMF.

It is noted that different solvents have been used for the DPV measurements. DCM is better for electrochemical characterization since it is easier to get rid of moisture compared to DMF. However, PorO faces solubility problem in DCM possibly due to the more planar structure of the carbazole fused-thiophene. Therefore, the characterization of PorO, PorR and their organic counterparts was used DMF as solvent.

The change of solvents from DCM to DMF resulted in a 0.1 eV variation of reduction potentials on Por (Por to Por#). This solvent effect is also observable for the di-chromophoric porphyrins, where the first reduction potential of PorO and the second reduction potential of PorR show 0.1 eV negative compared to the first reduction potential of PorY.

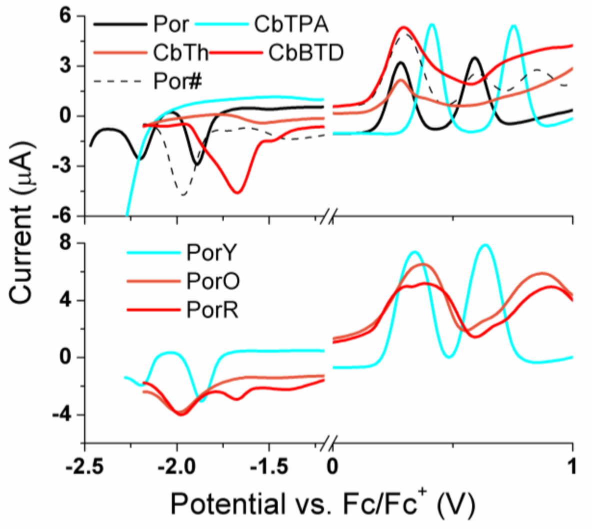

Figure 2. Differential pulse voltammograms of the compounds. Por, CbTPA and PorY were measured in DCM, while CbTh, PorO, CbBTD and PorR were measured in DMF.

Please do not adjust margins

Please do not adjust margins

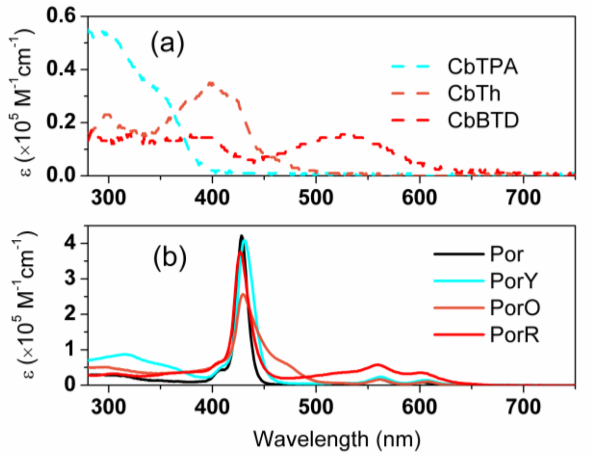

Figure 3. UV-visible absorption spectra of (a) the organic components and (b) the di-chromophoric porphyrins in solution with 1 μM concentration.

Fig.3 displays the UV-visible absorption spectra of all the compounds in DMF. As indicated by Fig.3(a), the UV-vis absorption of the three organic compounds are gradually red shifted from CbTPA to CbTh and to CbBTD, showing the onset wavelength of absorption from 400 nm to 450 nm and to 600 nm. Their peak molar extinction coefficients ε, however, reduce from 0.55×10^5 M^{-1} cm^{-1} of CbTPA to 0.34×10^5 M^{-1} cm^{-1} of CbTh and then to 0.16×10^5 M^{-1} cm^{-1} of CbBTD.

The UV-vis absorption spectrum of Por shows typical Zn porphyrin absorption features (Fig.3(b)): a strong Soret band with the peak molar extinction coefficient of 4.2×10^5 M^{-1} cm^{-1} at 428 nm and the modest Q band absorption and in the UV region with molar extinction coefficient of 0.4×10^5 M^{-1} cm^{-1}. The absorption spectra of the three di-chromophoric porphyrins show the feature of both Por and organic chromophore absorptions (Fig.3(b)). As compared to Por, PorY shows increased absorbance at the 280-400 nm wavelength region, PorO shows an extended absorption at the red edge of the Soret band to 500 nm and PorR shows an enhanced absorption from 460 nm to 640 nm, respectively, which are mainly due to the additional absorption by the organic chromophores.

It is observed that the absorption spectra of PorY, PorO and PorR closely resemble the superposition of the absorption spectra of their individual chromophore. This suggests the non-conjugated nature of these di-chromophoric dyes, which is a different molecular engineering approach from those conjugated sensitizers with strong push-pull effect by introducing functional groups with electron donating and electron withdrawing abilities; the latter approach is often observed a red-shift in light absorption spectrum.[24,25] An ~5 nm broadened Soret band is also observed in the di-chromophoric dyes when compared to Por, which is due to the effect of geometric distortion of the porphyrins.[13,26]

Steady state photoluminescence measurements were performed in solution at three different excitation wavelengths, (280 nm, 480 nm and 560 nm), the latter two selectively exciting each of the different chromophores. Fig.4 shows the photoluminescence spectra of the investigated compounds in solution. Normalized PL intensity with arbitrary unit is shown in Fig. S1.

PL of Por and organic chromophores at three different excitation wavelengths (Fig.4(a))

Photo-excitation of Por at 280 nm leads to two PL bands between 570 nm and 700 nm, which were assigned to the radiative decay from S_1 to the ground state S_0. Such a spectrum is characteristic of a Zn porphyrin chromophore reported in literature.[27,28] The emission from CbTPA being excited at 280 nm is seen in the wavelength range 420-700 nm. It is noted that this range overlaps with part of the Soret band and the entire Q band absorption of Por (Fig.3). The peak PL emission intensity of CbTPA is about 4 times higher compared to that of Por, whereas the absorption of CbTPA is only 2 times higher compared to that of Por at 280 nm (Fig.3), which indicates that the organic chromophore is a stronger emitter compared to the porphyrin. Photo-excitation of CbTh at 480 nm results in a PL band at 530 nm, which also covers the entire Q band absorption of Por. The PL band of CbBTD appears in the 580-800 nm wavelength range when photo-excited by either 480 nm or 560 nm.

PL of PorY, PorO and PorR at three different excitation wavelengths (Fig.4(b))

Photo-excitation of PorY leads to weak PL bands at 350-480 nm with peak intensity value of 0.05×10^5 CPS assigned to emission of Y unit. In addition, relatively strong bands with peak intensity value of 1.4×10^5 CPS at 600-700 nm assigned to the porphyrin emission are observed.

When PorO is photo-excited at 480 nm, the emission from the organic chromophore O at 530 nm is much weaker compared to that of CbTh, whereas the emission assigned to the porphyrin core at 600-720 nm wavelength range shows a peak value of 6×10^5 CPS. Note that the absorption of porphyrin core at 480 nm is marginal as indicated by Fig.3 and the emission of Por at 480 nm photo-excitation is negligible (Fig. 4(b)), the appearance of porphyrin emission in PorO suggests a substantial FRET from the organic chromophore O to the porphyrin core.

The PL band of PorR shows similar shape at photo-excitation of both 480 nm and 560 nm. It is noted that the porphyrin core in PorR only weakly absorbs photons at 480 nm; this similar PL band from the emission of PorR at two excitation wavelengths indicates that the emission of PorR originates mainly from the organic chromophore R. Compared to that of CbBTD in Fig.4(a), the PL intensity of PorR shows a three-fold greater emission when photo-excited at 560 nm. This difference in PL intensity is attributed to the differences in the absorption coefficients of PorR (0.58×10^5 M^{-1} cm^{-1}) and CbBTD (0.12×10^5 M^{-1} cm^{-1}) at the excitation wavelength (Fig.3).

This journal is © The Royal Society of Chemistry 20xx

Please do not adjust margins

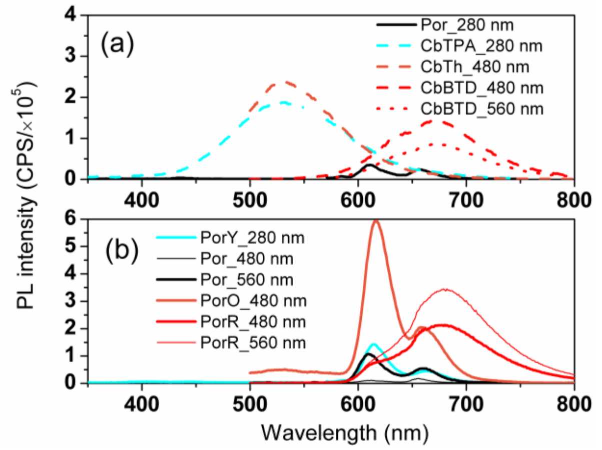

Figure 4. Photoluminescence (PL) spectra of (a) Por and the organic components and (b) the di-chromophoric porphyrins excited at specified wavelength with 1 μM concentration. Por, CbTPA, PorY, CbTh and PorO were measured in DMF, CbBTD and PorR were measured in THF.

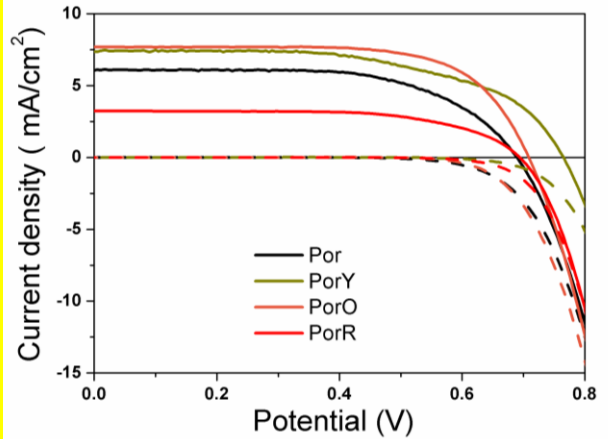

Figure 5. Current density-voltage (J-V) curves of DSSCs with the dyes fabricated using thin films under AM 1.5 illumination (solid lines) and in the dark (dashed lines).

Current density-voltage (J-V) curves of Por, PorY, PorO and PorR-sensitized solar cells under AM 1.5 illumination (solid lines) and in the dark (dashed lines) are shown in Fig.5. The photovoltaic parameters of these DSSCs are listed in Table 1.

The short circuit current density (J_{SC}) and open circuit voltage (V_{OC}) between Por and di-chromophoric porphyrins are varied. PorY and PorO achieved much greater photocurrent as compared to Por (7.0 and 7.4 mA cm^{-2} vs. 6.1 mA cm^{-2}, respectively), whereas PorR obtained quite moderate photocurrent, only around half value of Por. For the photovoltage, PorY accomplished the highest value of 770 mV, while PorO and PorR obtain slight larger values than Por. The power conversion efficiency of these DSSCs followed a similar trend with the photocurrent, which is that PorY and PorO performed the highest, followed by Por, and PorR the lowest.

The enhanced photovoltage of PorY and PorO-sensitized solar cells is due to the employing of the non-conjugated linker between the organic chromophore and the porphyrin, adding steric effect thus increasing the electron lifetime.[8,13] This bulky effect might also be applicable in PorR-sensitized solar cells as compared to Por-sensitized solar cells, however, previous measurements under reduced dye loading condition suggests that the strong dispersion forces between the organic chromophore R and the redox couple in electrolyte contribute significant to the longer electron lifetime of the former.[9]

The change in photocurrent of these DSSCs has been analyzed individually using the incident photon-to-current conversion efficiency (IPCE)-light harvesting efficiency (LHE)-absorbed photon-to-current conversion efficiency (APCE) approach in our previous studies.[9,13] However, the impact of energy / electron transfer between the two chromophores on electron injection has not been studied. Since electron injection efficiency of DSSC is roughly same as the APCE value when using thin film electrode where charge collection efficiency is uniform, APCE of DSSCs using the investigated porphyrins are measured as shown in Fig.6 and averaged APCE values at Soret and Q bands of the individual dyes are listed in Table 1.

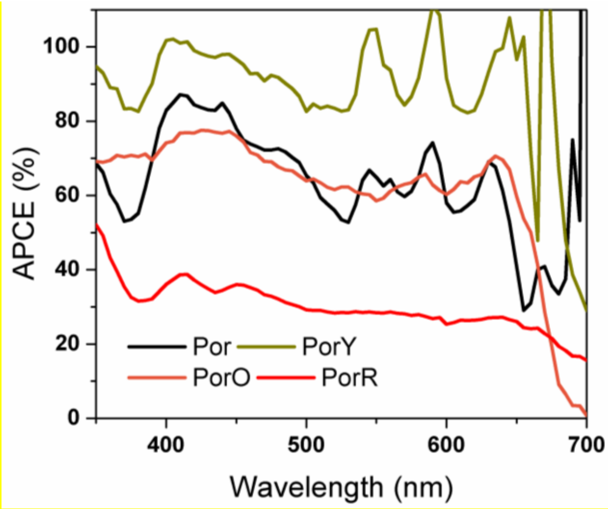

Figure 6. Absorbed photon-to-current conversion efficiency (APCE) spectra of the investigated dyes on TiO_2 thin films.

Table 1. Photovoltaic performance and averaged APCE values of DSSCs with the dyes fabricated using thin films

**Dye**	**J_{SC} mA cm^{-2}**	**V_{OC} mV**	**FF**	**PCE %**	**Averaged APCE Value (%)**	
					**Soret Band (400~450 nm)**	**Q Bands (550~650 nm)**
**Por**	6.1	690	0.62	2.6	85	60
**PorY**	7.0	770	0.60	3.3	95	90
**PorO**	7.4	700	0.69	3.6	75	60
**PorR**	3.2	700	0.63	1.4	35	30

Please do not adjust margins

The APCE values of DSSC in the Q band absorption region, when using PorY is 90%, significantly higher compared to when Por is employed (60%). The APCE values of DSSC using PorO are 60% in the Q band absorption region, which is very similar to that of DSSCs using Por. The broad shoulder between 450 nm and 500 nm is attributed to photo-excitation of the organic chromophore. PorR shows the lowest averaged APCE values at both Soret band and Q bands amongst these sensitizers, 35% and 30%, respectively, which is consistent with the trend of J_{SC}.

Discussion

1. Energy levels of the di-chromophoric porphyrins

An energy level diagram containing all the compounds obtained from the peak potentials in the DPV plots (Fig.2) is shown in Fig.7.

The energy gap of the second organic chromophores shows a decreasing trend, from 2.99 eV of CbTPA to 2.52 eV of CbTh and to 1.93 eV of CbBTD (Fig.7(a)). The first reduction potential (E_{Re}^{1st}) of the organic components CbTPA, CbTh and CbBTD, show a gradually more positive (easier to reduce) values, i.e. -2.53 eV, -2.86 eV and -3.44 eV vs Fc/Fc^+, respectively. The first oxidation potential (E_{Ox}^{1st}) of these organic components, on the other hand, shows a similar energy level at -5.40 eV vs Fc/Fc^+.

The E_{Re}^{1st} of Por, PorY and PorO are quite similar to each other at -3.20 eV vs Fc/Fc^+ , which is assigned to the reduction of the porphyrin core (Fig.7(b)). The E_{Re}^{1st} of PorR, on the other hand, is positively shifted by 0.20 eV compared to the other porphyrins and appears at similar potential as that of the CbBTD chromophore in Fig.7(a). Therefore, unlike those of the other porphyrins, the first reduction of PorR is assigned to the reduction of the organic chromophore R. The low-lying reduction potential of the organic chromophore R in PorR enables a possible intramolecular electron transfer from the photo-excited porphyrin to the chromophore with the electron acceptor character of the benzothiadiazole unit.

The E_{Ox}^{1st} of Por, PorY, PorO and PorR is within 0.10 eV variation of each other (-5.40 eV vs Fc/Fc^+). In fact, all the first oxidation peaks of the di-chromophoric sensitizers are comprised of a two-electron transfer reaction, forming a broad and intense current response plot (Fig.2). Spectro-electrochemical (SEC) measurements were carried out in the same solution as that of the electrochemical characterization, in order to distinguish these first oxidation peaks, as shown in Fig. S2. These SEC experiments however suggest that for the di-chromophoric sensitizers, distinguishing the origin of the E_{Ox}^{1st} would be difficult. The sensitizers are more likely to change to more planar structures after oxidation as compared to those in neutral state.

2. Energy / electron transfer pathway at dye-sensitized films

As indicated by the PL spectra in Fig.4, energy transfer from the organic chromophore to the porphyrin is possible in both PorY and PorO since the requirements of FRET are satisfied.[14,29,30] In PorY, both Por and Y chromophore are photo-excited at 280 nm. Energy transfer from Y* to Por increases the concentration of Por*, therefore enhancing emission from Por* and reducing emission from Y* to nearly unobservable. Similar to PorO, the marginal emission from O* as well as the strong emission from the Por at 480 nm photo-excitation both imply the energy transfer from O* to Por. However, energy transfer from the organic chromophore R to the porphyrin core does not likely occur in PorR. When photo-excited at 480 nm, there is no observable PL band assigning to the porphyrin from PorR emission as shown in Fig.4(b). In addition, energy transfer from the porphyrin to the organic chromophore in PorR is also not favored as suggested by the similar PL intensity of PorR and CbBTD after considering the absorbance of two molecules in THF at 560 nm. The emission quenched of Por in PorR when photo-excited at 560 nm, therefore, possibly originates from a photo-induced electron transfer pathway with a non-radiative back electron transfer as proposed below.

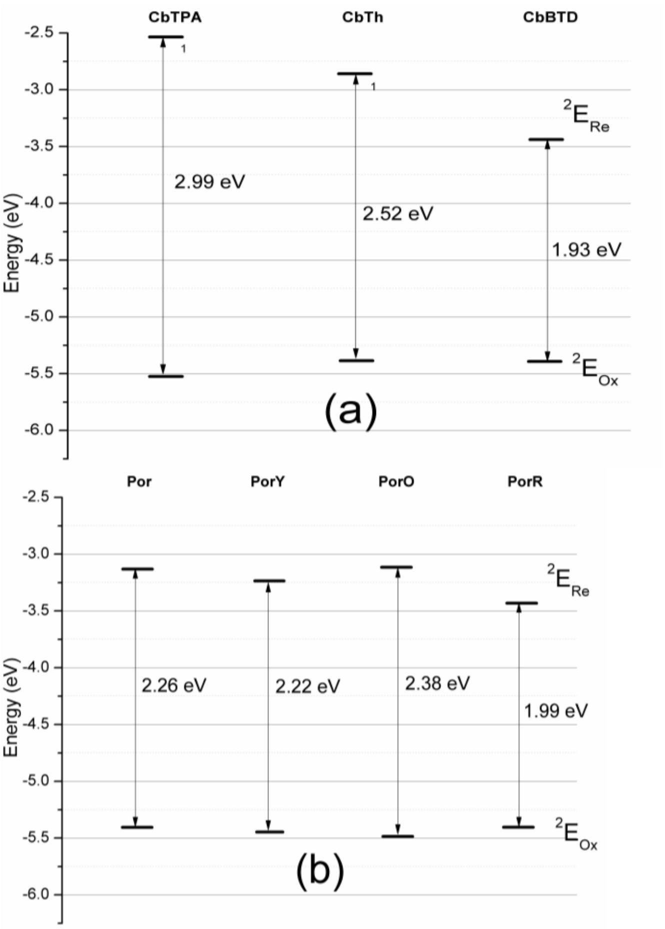

Figure 7. Energy level diagram of (a) the second organic chromophores and (b) the porphyrins derived from the peak potentials of differential pulse voltammograms. [1] Energy levels calculated from UV-vis absorption spectra by the equation $E_{Re}=\Delta E+E_{Ox}$, where the energy gap (ΔE) can get from the absorption of onset wavelength (λ_{onset}) by the equation $\Delta E=1243/\lambda_{onset}$. [2] The energy levels of oxidation (E_{Ox}) and the energy levels of reduction (E_{Re}) were calculated from the peak potentials of oxidation ($E_{Ox\ vs.\ Fc/Fc^+}$) and reduction ($E_{Re\ vs.\ Fc/Fc^+}$) as E_{Ox} = - ($E_{Ox\ vs.\ Fc/Fc^+}$ +5.1) (eV) and E_{Re} = - ($E_{Re\ vs.\ Fc/Fc^+}$ +5.1) (eV), respectively. The potential of the Fc/Fc^+ redox couple was assumed to be -5.1 eV vs. vacuum.[31]

This journal is © The Royal Society of Chemistry 20xx

Please do not adjust margins

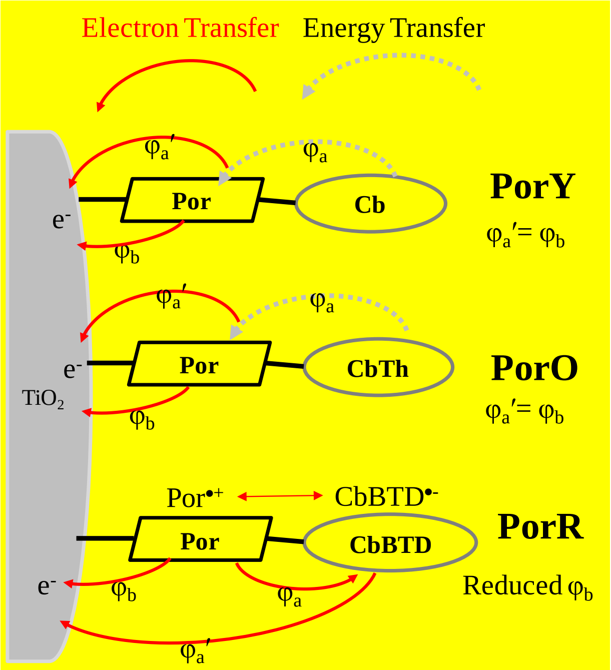

Figure 8. Electron / energy transfer pathways of the investigated dyes on TiO_2 thin films.

When photo-excited at 560 nm, the electron transfer from the porphyrin core to the organic chromophore in PorR may lead to an oxidized porphyrin cation radical ($Por^{•+}$) and a reduced carbazole-thiophene benzothiadiazole thiophene moiety ($CbBTD^{•-}$). The two ions might form an exciplex ($Por^{•+}$•$CbBTD^{•-}$)* under a PET mechanism or electron exchange (Dexter mechanism). The non-radiative back electron transfer ($Por^{•+}$ ↔ $CbBTD^{•-}$) results in no observation of PL from Por* in PorR in Fig.4(b). The distance between the absorber (Por) and the quencher (CbBTD or R) allows the efficient electron transfer efficiency since they are in one molecule.

In addition, emission spectra of both PorR and CbBTD was measured in DMF solvent under the same experimental condition. Compared to that in THF solvent, PL intensity of both PorR and CbBTD in DMF are much weaker (Fig.S3). Such a strong solvent effect suggests charge transfer nature of the emissive state in both molecules: in DMF with stronger polarity, charge transfer state is more stabilized leading to less emission, compared to the less polar THF.[32] Hence, PET pathway is proposed here as the main approach that quenches the Por emission in PorR.

It should be pointed that identifying the contributions of FRET or electron transfer for the PL quenching requires detailed measurements on PL lifetimes, quantum yields and understanding in electron transfer mechanism.[33,34] While these investigations are quite demanding on both instrument and elaboration, the work present here provides the primary study and correlates the possible energy / electron transfer mechanism to electron injection efficiency in DSSCs.

The proposed energy / electron transfer pathways at the three di-chromophoric porphyrin-sensitized TiO_2 films is shown schematically in Fig.8. Energy transfer from the organic chromophore to the porphyrin, in either PorY or PorO sensitized TiO_2, is expected to contribute to charge generation at wavelengths where the organic chromophore are selectively excited. PET from the porphyrin to the organic chromophore in PorR sensitized TiO_2, on the other hand, may decrease the charge injection efficiency leading to lower APCE.

The more significant APCE values of DSSC using PorY as compared to that of Por has been attributed to increased injection efficiency due to increased bulkiness of PorY and lower concentration of "non-injecting dye".[13] Since porphyrin, electrolyte and TiO_2 all absorb at around 400 nm, selective photo-excitation of the organic chromophore in DSSC is not feasible. Therefore, it is not possible to evaluate the effect of energy transfer from the photo-excited Y to Por. The relative values of APCE below 400 nm did not decrease for DSSCs sensitized with PorY compared to that of Por (Fig.6), which suggest that energy transfer does lead to photo-generation with the same overall quantum yield ($\phi_a'=\phi_b$, PorY in Fig.8).

A broad shoulder between 450 nm and 500 nm in APCE of PorO-sensitized solar cell is attributed to photo-excitation of the organic chromophore O. The APCE values at this wavelength range are lower compared to that at the Soret band, but on average, higher than that at the Q bands suggesting similarly efficient photo-generation from the photo-excited organic chromophore compared to the photo-excited porphyrin ($\phi_a'=\phi_b$, PorO in Fig.8). From this, it is not possible to distinguish between an energy transfer step from the O to the porphyrin core, followed by electron injection from the porphyrin or direct electron injection from the organic chromophore. However, the injection quantum yield from the two chromophores in PorO is similar.

The low APCE at the Soret band in DSSC using PorR suggests an intense decreased electron injection efficiency, which is quite possible, as discussed above, due to electron transfer to the organic chromophore, competes with electron injection into the conduction band of TiO_2 from the excited porphyrin core (decreased ϕ_b of PorR in Fig.8). Additionally, the APCE values were even lower at wavelengths above 660 nm, where the organic chromophore absorbs (Fig.6). This suggests that the charge generation efficiency of PorR is relatively lower compared to that of Por (electron transfer with efficiency of ϕ_a' of PorR in Fig.8). The poor photocurrent of PorR-sensitized solar cell in Fig.5 is also assigned to this compete electron transfer pathway.

3. Challenge in multi-chromophoric sensitizer design

The low APCE of PorR-sensitized DSSC is not due to some other effects such as dye aggregation,[35,36] the conduction band edge potential of TiO_2 being too high (diminishing the driving force for electron injection)[37] or sub-nanosecond recombination between the oxidized dye and surface trapped electrons.[38,39] The electron injection efficiency of a di-chromophoric dye-sensitized electrode is similar to that using chenodeoxycholic acid (CDCA) as a co-adsorber in Por-sensitized electrode, which suggests that rational designed di-chromophoric dyes may intrinsically prevent aggregation since their non-conjugated property.[13] Some other organic sensitizers with twisted structure also suggested an aggregation-free property.[40] As investigated by stepped-light induced measurements

Please do not adjust margins

of photocurrent and photovoltage (SLIM-PCV), the TiO_2 CB edge potential of PorR-sensitized DSSC was identical to that of Por-sensitized DSSC, which suggests that the driving forces for electron injection of the two systems are similar.[9] The fast recombination component at the dye-sensitized semiconductor surface is assigned to the strong coupling between the surface trap state and the oxidized dye,[41] which should not be the cause in PorR-sensitized TiO_2 electrode since firstly, that all the three di-chromophoric dyes PorY, PorO and PorR are used the same porphyrin structure with typical less than 200 femtoseconds electron injection kinetics as coupling to the TiO_2 conduction band,[39] and secondly, that steady state photoluminescence studies in Fig. 4 suggest similar photoluminescence intensity within the three di-chromophoric dyes, which may suggest similar excited state lifetime.

The observations and discussions above proposed a major challenge in designing multi-chromophoric dyes for application in DSSCs. To increase the light absorption of sensitizers, chromophores with narrow frontier orbital energy offsets are good candidates, while the position of these chromophores in multi-chromophoric dye molecules needs to be carefully considered. Attaching such a chromophore further away of the TiO_2 surface, like in the case of PorR dye, can significantly reduce the power conversion efficiency as this opens a competitive charge transfer pathway. This may lead to poor photocurrent generation in a DSSC due to the insufficient electron injection or low photovoltage as a result of the strong intermolecular forces at the TiO_2/dye/electrolyte interface.[42,43]

One possible approach to solve this problem is to covalently attached the low energy gap chromophore further away from the TiO_2 surface and to better couple the excited state of the low energy gap chromophore with the conduction band electrons in TiO_2. In this way, remote electron injection from the red shifted chromophore would increase, whilst the recombination between the injected electrons with the electrolyte would be harder since the larger dispersion forces between the low energy gap chromophore and the electrolyte. Another possible approach would be to design a low energy gap chromophore with high electron injection efficiency and to tailor the multi-chromophoric dyes with long-live charge separation.[44,45] These approaches, albeit accompany with a few trade-offs, would be interesting research areas for novel dye molecule engineering for advanced photocurrent conversion devices.

Conclusion

Three di-chromophoric dyes containing a porphyrin and an organic chromophore with gradually decreased energy levels were characterized both in solution and on TiO_2 surface. The energy / electron transfer mechanisms within the two chromophores in three di-chromophoric porphyrins have been characterized using steady state photoluminescence spectroscopy measurements in concert with the absorbed photon-to-current conversion efficiency measurements. Similar charge generation efficiency from the two chromophores was suggested in PorY and PorO sensitized TiO_2 electrodes, in which energy transfer occurred from the organic chromophore to the porphyrin. When a low energy gap organic chromophore was introduced to the porphyrin-based di-chromophoric sensitizer, electron transfer, rather than an energy transfer, from the excited porphyrin to the organic chromophore was strongly suggested. This electron transfer pathway in PorR-sensitized TiO_2 film competes with electron injection from the porphyrin to TiO_2 conduction band, leading to a low APCE of only 30%. These findings about the charge generation mechanism demonstrate the possible limitations in multi-chromophoric sensitizers and provide valuable information for the next generation of multi-chromophoric dye molecule structure design.

RSOS-end-sections

Ethics. There is no requirement to complete research / animal ethical assessments prior to conducting this research. There is no permissions on carrying out fieldwork being required prior to conducting this research.

Data accessibility. All data supporting the findings of this study are included in the manuscript and supporting information file.

Competing interests. I have no competing interests.

Funding. The author acknowledges the Natural Science Foundation of China (No. 21703086), China Postdoctoral Science Foundation (No. 2017M611706) and a matching scholarship from University of Wollongong.

Acknowledgements. LZ wishes to thank A. J. Mozer, A. Nattestad, P. Wagner, T. M. Clarke and S. Mori for discussions.

References

(1) Mathew, S.; Yella, A.; Gao, P.; Humphry-Baker, R.; Curchod, B. F. E.; Ashari-Astani, N.; Tavernelli, I.; Rothlisberger, U.; Nazeeruddin, M. K.; Grätzel, M. *Nat. Chem.* **2014**, *6*, 242.
(2) Yella, A.; Lee, H.-W.; Tsao, H. N.; Yi, C.; Chandiran, A. K.; Nazeeruddin, M. K.; Diau, E. W.-G.; Yeh, C.-Y.; Zakeeruddin, S. M.; Grätzel, M. *Science* **2011**, *334*, 629.
(3) Li, L.-L.; Diau, E. W.-G. *Chem. Soc. Rev.* **2013**, *42*, 291.
(4) Campbell, W. M.; Burrell, A. K.; Officer, D. L.; Jolley, K. W. *Coord. Chem. Rev.* **2004**, *248*, 1363.
(5) Imahori, H. *J. Phys. Chem. B* **2004**, *108*, 6130.
(6) Mozer, A. J.; Griffith, M. J.; Tsekouras, G.; Wagner, P.; Wallace, G. G.; Mori, S.; Sunahara, K.; Miyashita, M.; Earles, J. C.; Gordon, K. C.; Du, L.; Katoh, R.; Furube, A.; Officer, D. L. *J. Am. Chem. Soc.* **2009**, *131*, 15621.
(7) Warnan, J.; Pellegrin, Y.; Blart, E.; Odobel, F. *Chem. Commun.* **2012**, *48*, 675.
(8) Zhao, L.; Wagner, P.; van der Salm, H.; Clarke, T. M.; Gordon, K. C.; Mori, S.; Mozer, A. J. *J. Phys. Chem. C* **2015**, *119*, 5350.
(9) Zhao, L.; Wagner, P.; van der Salm, H.; Gordon, K. C.; Mori, S.; Mozer, A. J. *ACS Appl. Mater. Interfaces* **2015**, *7*, 22078.
(10) Mai, C.-L.; Huang, W.-K.; Lu, H.-P.; Lee, C.-W.; Chiu, C.-L.; Liang, Y.-R.; Diau, E. W.-G.; Yeh, C.-Y. *Chem. Commun.* **2010**, *46*, 809.
(11) Watson, B. L.; Moore, T. A.; Moore, A. L.; Gust, D. *Dyes and Pigments* **2017**, *136*, 893.
(12) Zhao, L.; Wagner, P.; Barnsley, J. E.; Clarke, T. M.; Gordon, K. C.; Mori, S.; Mozer, A. J. *Chem. Sci.* **2016**, *7*, 3506.

This journal is © The Royal Society of Chemistry 20xx

Please do not adjust margins

Please do not adjust margins

(13) Zhao, L.; Wagner, P.; Elliott, A. B. S.; Griffith, M. J.; Clarke, T. M.; Gordon, K. C.; Mori, S.; Mozer, A. J. *J. Mater. Chem. A* **2014**, *2*, 16963.
(14) Basham, J. I.; Mor, G. K.; Grimes, C. A. *ACS Nano* **2010**, *4*, 1253.
(15) Williams, R. M. *University of Amsterdam, PhD thesis* **1996**.
(16) Hardin, B. E.; Sellinger, A.; Moehl, T.; Humphry-Baker, R.; Moser, J.-E.; Wang, P.; Zakeeruddin, S. M.; Grätzel, M.; McGehee, M. D. *J. Am. Chem. Soc.* **2011**, *133*, 10662.
(17) Murphy, C. B.; Zhang, Y.; Troxler, T.; Ferry, V.; Martin, J. J.; Jones, W. E. *J. Phys. Chem. B* **2004**, *108*, 1537.
(18) Hirakawa, K.; Segawa, H. *J. Photochem. Photobiol. A. Chem.* **1999**, *123*, 67.
(19) Zhou, J.; Shah, R. P.; Findley, B. R.; Braun, C. L. *J. Phys. Chem. A* **2002**, *106*, 12.
(20) Song, H.-e.; Taniguchi, M.; Diers, J. R.; Kirmaier, C.; Bocian, D. F.; Lindsey, J. S.; Holten, D. *J. Phys. Chem. B* **2009**, *113*, 16483.
(21) Odobel, F.; Pellegrin, Y.; Warnan, J. *Energy Environ. Sci.* **2013**, *6*, 2041.
(22) Argazzi, R.; Bignozzi, C. A.; Heimer, T. A.; Meyer, G. J. *Inorg. Chem.* **1997**, *36*, 2.
(23) Kubo, W.; Sakamoto, A.; Kitamura, T.; Wada, Y.; Yanagida, S. *J. Photochem. Photobiol., A* **2004**, *164*, 33.
(24) Koumura, N.; Wang, Z.-S.; Mori, S.; Miyashita, M.; Suzuki, E.; Hara, K. *J. Am. Chem. Soc.* **2006**, *128*, 14256.
(25) Wu, S.-L.; Lu, H.-P.; Yu, H.-T.; Chuang, S.-H.; Chiu, C.-L.; Lee, C.-W.; Diau, E. W.-G.; Yeh, C.-Y. *Energy Environ. Sci.* **2010**, *3*, 949.
(26) Kim, D.; Osuka, A. *Acc. Chem. Res.* **2004**, *37*, 735.
(27) Lin, C.-Y.; Lo, C.-F.; Luo, L.; Lu, H.-P.; Hung, C.-S.; Diau, E. W.-G. *J. Phys. Chem. C* **2009**, *113*, 755.
(28) Xue, X.; Zhang, W.; Zhang, N.; Ju, C.; Peng, X.; Yang, Y.; Liang, Y.; Feng, Y.; Zhang, B. *RSC Adv.* **2014**, *4*, 8894.
(29) Förster, T. *Discuss. Faraday Soc.* **1959**, *27*, 7.
(30) Jares-Erijman, E. A.; Jovin, T. M. *Nat Biotech* **2003**, *21*, 1387.
(31) Cardona, C. M.; Li, W.; Kaifer, A. E.; Stockdale, D.; Bazan, G. C. *Adv. Mater.* **2011**, *23*, 2367.
(32) Debnath, T.; Maity, P.; Lobo, H.; Singh, B.; Shankarling, G. S.; Ghosh, H. N. *Chem. Eur. J.* **2014**, *20*, 3510.
(33) Lazarides, T.; Kuhri, S.; Charalambidis, G.; Panda, M. K.; Guldi, D. M.; Coutsolelos, A. G. *Inorg. Chem.* **2012**, *51*, 4193.
(34) Algar, W. R.; Khachatrian, A.; Melinger, J. S.; Huston, A. L.; Stewart, M. H.; Susumu, K.; Blanco-Canosa, J. B.; Oh, E.; Dawson, P. E.; Medintz, I. L. *Journal of the American Chemical Society* **2017**, *139*, 363.
(35) Sunahara, K.; Furube, A.; Katoh, R.; Mori, S.; Griffith, M. J.; Wallace, G. G.; Wagner, P.; Officer, D. L.; Mozer, A. J. *J. Phys. Chem. C* **2011**, *115*, 22084.
(36) Lim, J.; Kwon, Y. S.; Park, T. *Chem. Commun.* **2011**, *47*, 4147.
(37) Katoh, R.; Furube, A.; Barzykin, A. V.; Arakawa, H.; Tachiya, M. *Coord. Chem. Rev.* **2004**, *248*, 1195.
(38) Imahori, H.; Kang, S.; Hayashi, H.; Haruta, M.; Kurata, H.; Isoda, S.; Canton, S. E.; Infahsaeng, Y.; Kathiravan, A.; Pascher, T.; Chabera, P.; Yartsev, A. P.; Sundstrom, V. *J. Phys. Chem. A* **2011**, *115*, 3679.
(39) Griffith, M. J.; Sunahara, K.; Wagner, P.; Wagner, K.; Wallace, G. G.; Officer, D. L.; Furube, A.; Katoh, R.; Mori, S.; Mozer, A. J. *Chem. Commun.* **2012**, *48*, 4145.
(40) Numata, Y.; Islam, A.; Chen, H.; Han, L. *Energy Environ. Sci.* **2012**, *5*, 8548.
(41) Johansson, P. G.; Kopecky, A.; Galoppini, E.; Meyer, G. J. *J. Am. Chem. Soc.* **2013**, *135*, 8331.
(42) Zhou, W.; Cao, Z.; Jiang, S.; Huang, H.; Deng, L.; Liu, Y.; Shen, P.; Zhao, B.; Tan, S.; Zhang, X. *Org. Electron.* **2012**, *13*, 560.
(43) Watson, B. L.; Sherman, B. D.; Moore, A. L.; Moore, T. A.; Gust, D. *Phys. Chem. Chem. Phys.* **2015**, *17*, 15788.
(44) Ardo, S.; Meyer, G. J. *Chem. Soc. Rev.* **2009**, *38*, 115.
(45) Fukuzumi, S.; Ohkubo, K.; Suenobu, T. *Acc. Chem. Res.* **2014**, *47*, 1455.

Please do not adjust margins
