# Supplementary material for: Effect of the second chromophore energy gap on photo-induced electron injection in di-chromophoric porphyrin-sensitized solar cells
Source: R Soc Open Sci. 2018 Sep 26;5(9):181218. doi: 10.1098/rsos.181218 (PMC6170585; doi:10.1098/rsos.181218)
Supplement: Maintext-R1 for rsos-hightlight changes [file rsos181218supp2.docx]

# Supporting information

# Effect of the second chromophore energy gap on photo-induced electron injection in di-chromophoric porphyrin-sensitized solar cells

Long Zhao*

School of Chemistry and Chemical Engineering, Jiangsu University, Zhenjiang, 212013, P. R. China; ARC Centre of Excellence for Electromaterials Science, Intelligent Polymer Research Institute, University of Wollongong, Wollongong, NSW 2522, Australia.

Figure S1. Normalized photoluminescence spectra of (a) Por and the organic components and (b) the di-chromophoric porphyrins excited at specified wavelength.

Figure S2. Spectro-electrochemical (SEC) spectra of (a) Por; (b) CbTPA; (c) CbTh; (d) CbBTD; (e) PorY; (f) PorO; (g) PorR in the same solution as Fig. S1 at different oxidation potentials vs. Fc/Fc^+^. The inset gives the DPV oxidation plots and indicates the applied potentials by colourful lines.

Spectroelectrochemical (SEC) spectroscopy was carried out in the same solution as DPV characterization for each compound in a optically transparent thin layer electrochemical (OTTLE) cell. Fig.S2 shows the SEC spectra of the investigated compounds under a series of potentials. The change in absorption (ΔAbs.) indicates the change in light absorption between the radical cation and the dye ground state. In the case where ΔAbs.>0, it indicates formation of dye radical cation. In the case where ΔAbs.<0, it suggests bleaching of dye ground state. The absorption of radical cations stemming from Por, CbTPA and CbBTD occurs at 0.35 V vs Fc/Fc^+^, while that of CbTh emerges at around 0.50 V vs Fc/Fc^+^. Similarly, the absorption of radical cations stemming from the three di-chromophoric dyes appears at 0.35 V vs Fc/Fc^+^. Note that the SEC spectra of PorO seems to be a combination ΔAbs. from both Por and CbTh, which is in consistence with the weak conjugation nature of PorO as suggested by both DPV results and DFT calculations in a previous report.[^1^](#_ENREF_1)

Figure S3. Photoluminescence intensities measured by photoexcitation of CbBTD and PorR dissolved in DMF with 1 µM concentration at 560 nm.

(1) Zhao, L.; Wagner, P.; van der Salm, H.; Clarke, T. M.; Gordon, K. C.; Mori, S.; Mozer, A. J. Dichromophoric Zinc Porphyrins: Filling the Absorption Gap between the Soret and Q Bands. *J. Phys. Chem. C* **2015**, *119*, 5350-5363.
